# In silico approaches for developing sesquiterpene derivatives as antagonists of human nicotinic acetylcholine receptors (nAChRs) for nicotine addiction treatment

**DOI:** 10.1016/j.crstbi.2024.100162

**Published:** 2025-01-02

**Authors:** Taufik Muhammad Fakih, Aden Dhana Rizkita, Sintia Ayu Dewi, Muchtaridi Muchtaridi

**Affiliations:** a Department of Pharmaceutical Analysis and Medicinal Chemistry, Faculty of Pharmacy, Universitas Padjadjaran, Jalan Raya Bandung-Sumedang KM 21, Sumedang, 45363, Indonesia; b Department of Pharmacy, Faculty of Mathematics and Natural Sciences, Universitas Islam Bandung, Jl. Ranggagading No.8, Bandung, 40116, Indonesia; c Department of Pharmacy, Sekolah Tinggi Ilmu Kesehatan (STIKES) Bogor Husada, Jl. Sholeh Iskandar No.4, Bogor, 16164, Indonesia; d Graduate Institute of Pharmacognosy, College of Pharmacy, Taipei Medical University, No. 250 Wu-Xing Street, Taipei, 11031, Taiwan; e Research Collaboration Centre for Radiopharmaceuticals Theranostic, National Research and Innovation Agency (BRIN), Jalan Raya Bandung-Sumedang KM 21, Sumedang, 45363, Indonesia

**Keywords:** Sesquiterpene compounds, Cinnamomum genus, Nicotinic acetylcholine receptors (nAChRs), Inhibitor nAChRÿ3, In silico approaches

## Abstract

Cinnamomum, a genus within the Lauraceae family, has gained global recognition due to its wide-ranging utility. Extensive research has been dedicated to exploring its phytochemical composition and pharmacological effects. Notably, the uniqueness of Cinnamomum lies in its terpenoid content, characterized by distinctive structures and significant biological implications. An intriguing discovery is that sesquiterpene compounds originating from Cinnamomum possess the capacity to function as antagonists for human nicotinic acetylcholine receptors (nAChRs), specifically the nAChRÿ3 subtype, rendering them potential candidates for nicotine replacement therapy (NRT) to aid active smokers. This investigation employed molecular docking and molecular dynamics simulations to assess the inhibitory effects of these compounds on nAChRÿ3. Among the 55 compounds examined, Dihydroxyeudesmene, Gibberodione, and Germacrene-E exhibited the highest binding affinities. These compounds demonstrated robust interactions with the nAChRÿ3 receptor, as evidenced by elevated molecular mechanics general surface area (MM/GBSA) values (ΔG Bind = Dihydroxyeudesmene: −36.45 kcal/mol, Gibberodione: −36.51 kcal/mol, and Germacrene-E: −36.51 kcal/mol). Molecular dynamics simulations further confirmed the stability of these three compounds, indicating their potential to effectively compete with native ligands. However, comprehensive in vitro, in vivo, and clinical investigations are imperative to ascertain the efficacy of these promising therapeutic candidates.

## Graphical abstract

**Figure:**
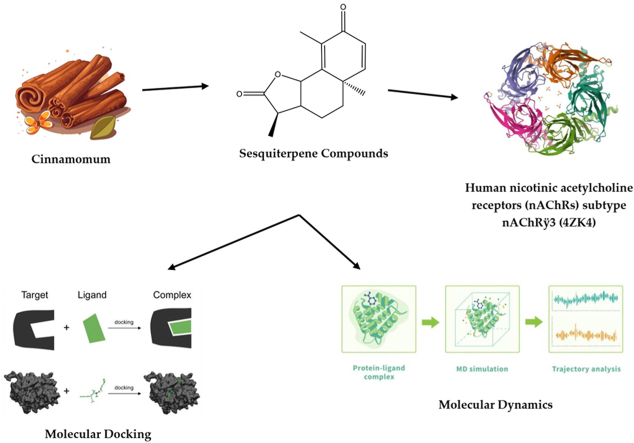

## 1 Introduction

Cinnamomum, part of the Lauraceae family, encompasses a group of evergreen trees and shrubs. This genus comprises roughly 250 species distributed across the globe, primarily inhabiting tropical and subtropical regions within Southeast Asia, Australia, as well as North, Central, and South America (Yang et al., 2022; Didehdar et al., 2022). In China, there are approximately 46 species, predominantly found in the southern regions, with Yunnan province boasting the highest number of species, followed by Guangdong and Sichuan. Cinnamomum holds significant recognition on a global scale owing to its versatile utility (Wu et al., 2022). Traditionally, it has been employed to impart flavor to culinary dishes and has been a staple in folk medicine due to its sweat-inducing, fever-reducing, and pain-relieving properties (Lee et al., 2022).

Numerous studies have delved into the phytochemistry of the Cinnamomum genus, with a primary focus on around 14 specific species (Zhang et al., 2017). These investigations have unveiled the presence of a rich and diverse array of terpenoids within the genus, boasting distinctive structural characteristics. To date, an impressive total of 181 terpenoids have been successfully isolated from various species within the Cinnamomum genus, encompassing monoterpenes, sesquiterpenes, diterpenes, and triterpenes (Song et al., 2020; Rabah et al., 2017). Among these compounds, 119 terpenoids have been identified in different Cinnamomum species, including but not limited to *Cinnamomum cassia*, *Cinnamomum wilsonii*, *Cinnamomum camphora*, *Cinnamomum glanduliferum*, *Cinnamomum subavenium*, *Cinnamomum zeylanicum*, *Cinnamomum osmophloeum*, *Cinnamomum inunctum*, *Cinnamomum philippinense*, *Cinnamomum kotoense*, *Cinnamomum burmannii*, *Cinnamomum parthenoxylon*, *Cinnamomum reticulatum*, and *Cinnamomum tenuifolium*, respectively (Balijepalli et al., 2017; Sharifi-Rad et al., 2021). Of these, 55 terpenoids, including sesquiterpenoids, were selected for this study based on their bioactivity, structural diversity, and relevance to nicotinic acetylcholine receptor (nAChR) modulation, with a particular emphasis on sesquiterpenes due to their previously reported interactions with nAChRs (Moujir et al., 2020; Salazar-Gómez et al., 2020).

It is noteworthy that a significant portion of sesquiterpene compounds derived from Cinnamomum possesses the potential to serve as Nicotine Replacement Therapy (NRT), aimed at mitigating nicotine addiction. Numerous studies have illuminated the fact that the majority of these sesquiterpene compounds exhibit properties conducive to supporting smoking cessation efforts (Development and evaluation of a, 2020; Chakraborty et al., 2014). To illustrate, some of these compounds have demonstrated their ability to influence the human nicotinic acetylcholine receptor (nAChR), a key player in the human nervous system's response to nicotine (Wibowo et al., 2022). As a result, these compounds represent promising candidates for effective nicotine replacements, offering a lower health risk profile compared to smoking. Furthermore, they have the capacity to assist individuals in curbing their smoking cravings, thereby facilitating the process of smoking cessation (Hossan et al., 2009).

**Fig. 1:**
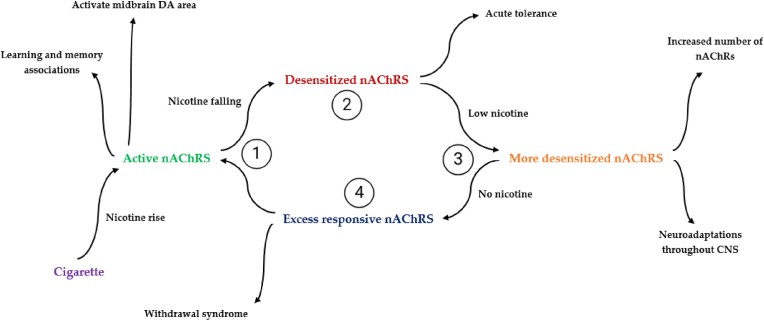
A straightforward pattern of ongoing tobacco consumption, driven by the cellular effects of nicotine (Dani and Heinemann, 1996).

Desensitization of nicotinic receptors has significant consequences (Fig. 1). When exposed to nicotine, particularly from tobacco, high-affinity nicotinic acetylcholine receptors (nAChRs) tend to undergo desensitization (Auerbach, 2020; Rahman et al., 2022). Desensitization occurs when prolonged or repeated exposure to agonists such as nicotine causes the receptors to transition into a non-responsive state. This is not due to a reduction in receptor numbers but rather a functional inactivation where the receptors are temporarily unable to open their ion channels, even in the presence of agonists (Cecchini and Changeux, 2022; Shen et al., 2016). This phenomenon is especially prominent at cholinergic synapses, where nAChRs are repetitively exposed to synaptic acetylcholine (ACh) as well as nicotine from cigarette smoke. Persistent activation by these agonists causes receptor conformational changes, rendering them unable to bind further or propagate signals, increasing the likelihood of desensitization at active synapses (Shen et al., 2022). This desensitization leads to a temporary reduction in the number of functional receptors available for normal synaptic signaling. Consequently, smoking diminishes the capacity of nicotinic cholinergic synapses to receive and process information, as fewer nAChRs can respond effectively to the release of ACh (Sun et al., 2020). Moreover, nicotine disrupts typical nicotinic cholinergic signaling while simultaneously sending inappropriate signals via the mesocorticolimbic dopamine system, contributing to both cognitive impairments and addiction (Matsuda, 2021).

In the process of antagonizing nAChRs, targeting the nAChRÿ3 subtype emerges as a promising strategy for addressing smoking addiction. This study aimed to identify potent inhibitors among sesquiterpene compounds derived from Cinnamomum. The selection of sesquiterpenes as the focus of this study is supported by prior evidence of their interaction with nicotinic acetylcholine receptors (nAChRs) in related systems (Rizkita et al., 2024). For instance, the monocyclic sesquiterpene alcohol bisabolol, found in the essential oil of several plants in the Asteraceae family and known for its anti-nociceptive and anti-inflammatory effects, was shown to directly bind and inhibit α7 nAChR-mediated currents (Nurulain et al., 2015). Additionally, drimane sesquiterpenoids such as drimenin, cinnamolide, dendocarbin A, and polygodial—purified from the Canelo tree (Drimys winteri) and characterized using spectroscopic methods—exhibited pharmacological activity on various nAChRs (Arias et al., 2018). These findings underscore the potential of sesquiterpenes as a promising class of natural compounds capable of modulating nAChR activity. Thus, the primary objective of this investigation was to identify nAChRÿ3 inhibitors among Cinnamomum compounds through computational methodologies.

## 2 Materials and methods

### 2.1 Ligand collection and preparation

In the initial phase of this investigation, we curated a collection of sesquiterpene compounds sourced from Cinnamomum based on relevant research findings and literature available in the PubMed, Google Scholar, Web of Science, and Scopus databases. Subsequently, we established a dataset comprising efficacious sesquiterpene compounds from Cinnamomum, which was extracted from the PubChem database (Kim et al., 2023). The molecular structures of these ligands were acquired and meticulously prepared using the ligand preparation tool within ChemDraw Professional 16.0 and Chem3D 16.0 (Brown, 2014). These structures were further optimized using quantum-mechanical (QM) methods with Density Functional Theory (DFT) (B3LYP/3-21G) basis set through Quantum ESPRESSO v.7.1 (Giannozzi et al., 2020), before proceeding to the subsequent molecular docking analysis.

### 2.2 Receptor preparation

The crystallographic structures of human nicotinic acetylcholine receptors (nAChRs) subtype nAChRÿ3 were obtained from the Protein Data Bank archive (PDB ID: 4ZK4), and these structures were subjected to refinement. This refinement process involved the removal of water molecules and optimization of the proteins under neutral pH conditions. To create the definitive receptor, the Amber ff14SB-ILDN algorithm, as implemented in SWISSPDB and Chimera viewers, was utilized, incorporating the Amber force field (Pettersen et al., 2004). Several modifications were made, which included adjustments to thiol and hydroxyl groups, amino groups found in asparagine and glutamine, and imidazole rings present in histidine. Additionally, protonation states of histidine, aspartic acid, and glutamic acid were considered. A final minimization step was carried out using the Amber ff14SB-ILDN force field, with the maximum allowable heavy atom RMSD set at 3.0 Å.

### 2.3 Molecular docking simulation

Molecular docking of all compounds was conducted using the MGLTools 1.5.7 and AutoDockTools 4.2.6 software modules (Forli et al., 2012). To identify compounds with the desired biological activity, a virtual screening process was employed, treating ligands as flexible entities and receptors as rigid structures during the docking procedure. Docking was performed using the Lamarckian Genetic Algorithm (LGA), with the following parameters: a population size of 150, a maximum number of 2,500,000 energy evaluations, and 27,000 generations per docking run (Mohapatra et al., 2015). Compounds were evaluated based on their binding energy and root mean square deviation (RMSD) values. Ligands with docking scores below the cutoff value of −8.50 kcal/mol and RMSD values less than 2.0 Å were shortlisted for further investigation. RMSD values provide insights into the precision of docking orientations, with values below 2.0 Å indicating reliable binding poses (Fakih, 2021). The clustering cutoff was set at 2.0 Å to group similar binding conformations, and rank 1 poses from the most populated clusters were selected by default for downstream analyses. Molecular interactions between ligands and receptors were visualized and analyzed using BIOVIA Discovery Studio Visualizer v 2019 Client (BIOVIA, 2005). These interactions included hydrogen bonding, hydrophobic interactions, and π-π stacking, offering detailed insights into the binding mechanisms.

### 2.4 Prime molecular mechanics-generalized born and surface area (MM-GBSA)

The Prime MM-GBSA module within the Schrodinger-Maestro v 9.4 suite was employed to compute the binding affinity (Kotni, 2015; Ahmad and Raisuddin, 2020). A more negative affinity value indicates a higher level of stability. The calculation was executed using a Glide XP docking file with the docked Pose viewer. A continuum model, the Generalized Born Surface Accessible (GBSA) method, was employed in the sample minimization process. Molecular mechanics (MM) with the Amber ff14SB-ILDN force field was used, allowing for the flexibility of the protein. VSGB 2.0 served as a dielectric solvent model to account for pi-stacking and H-bond empirical functions in interactions.

### 2.5 Molecular dynamics (MD) simulations

MD simulations were conducted using the GROMACS v 2016.3 program and the Amber ff14SB-ILDN force field (Aragones et al., 2013; Abraham et al., 2015; Lumbanraja et al., 2023). The Auto SMILE algorithm was employed to determine parameters for each ligand within the protein-ligand complexes. This process involved automatic parameterization of unidentified organic molecules, calculating semi-empirical AM1 Mulliken point charges with the COSMO solvation model, assigning atom and bond types using AM1BCC, and allocating GAFF atom types and other force field parameters (Fouda et al., 2012). Prior to the simulation, optimization and resolution of the protein-ligand complex's hydrogen bond network were carried out using the TIP3P water model in the simulation cell. In the solvation process, pKa calculations were performed based on the titratable amino acids present in the protein complex. Initial energy minimization was achieved using the simulated annealing method with the steepest gradient strategy (5000 cycles). Each simulation system consisted of approximately 62,521 ± 10 atoms. The simulations were conducted in a physiological environment (298 K, pH 7.4, 0.9 percent NaCl) with a time step of 2.0 fs as part of the multiple time step algorithm. All bond lengths were constrained using the LINCS (linear constraint solver) algorithm, while ETTLE was applied to water molecules. The PME approach was used to account for long-range electrostatic attraction. The MD simulation ran for 500 ns under constant pressure conditions with a Berendsen thermostat. Subsequently, MM-PBSA (MM Poisson-Boltzmann surface area) was used to calculate the binding free energies for all snapshots with the GROMACS v 2016.3 software (Ren et al., 2020).

## 3 Results

### 3.1 Virtual screening based on docking scores of sesquiterpene compounds from Cinnamomum

Virtual screening, a valuable method for molecular docking, is employed to identify potential lead compounds in drug discovery. In this study, we conducted molecular docking analysis involving 55 previously identified compounds, focusing on their interactions with human nicotinic acetylcholine receptors (nAChRs), specifically subtype nAChRÿ3. For nAChRÿ3, we selected the top three compounds based on their superior docking scores. These scores were determined using the MGLTools 1.5.7 and AutoDockTools 4.2.6 docking programs, with a set cutoff value of −8.50 kcal/mol. Consequently, the best docking scores were recorded as follows: Dihydroxyeudesmene displayed a score of −8.50 kcal/mol, Gibberodione obtained a score of −8.56 kcal/mol, and Germacrene-E also achieved a score of −8.56 kcal/mol.

### 3.2 MM-GBSA binding affinity estimation

To ensure a more robust estimation, MM-GBSA analysis was performed on the selected three compounds (n = 3), chosen based on their binding affinity calculations and interactions with the receptor catalytic pair in nAChRÿ3. The three ligands exhibiting the highest binding affinities (ΔG Bind) were identified as follows: Dihydroxyeudesmene with a binding affinity of −36.45 kcal/mol, Gibberodione with −36.51 kcal/mol, and Germacrene-E also with −36.51 kcal/mol. Given that the relative scores from MM-GBSA was nearly identical for these compounds, the efficacy of one over the others cannot be overstated. This similarity highlights the need for further validation to distinguish their functional potential. To address this, molecular dynamics (MD) simulations were conducted to evaluate the stability and dynamic behavior of the receptor-ligand complexes over time. MD simulations provide critical insights into the binding stability, conformational changes, and interaction profiles that cannot be captured through docking and MM-GBSA analysis alone (Serafeim et al., 2016). Before conducting docking experiments with sesquiterpene compounds sourced from Cinnamomum, the existing 7-(5-isopropoxy-pyridin-3-yl)-1-methyl-1,7-diaza-spiro[4.4]nonane ligand was reassembled as the native ligand with nAChRÿ3. The RMSD difference was approximately 1.90 Å, which is well within the acceptable range of 3.0 Å, ensuring the reliability of the docking poses.

### 3.3 Ligand binding analysis

**Fig. 2:**
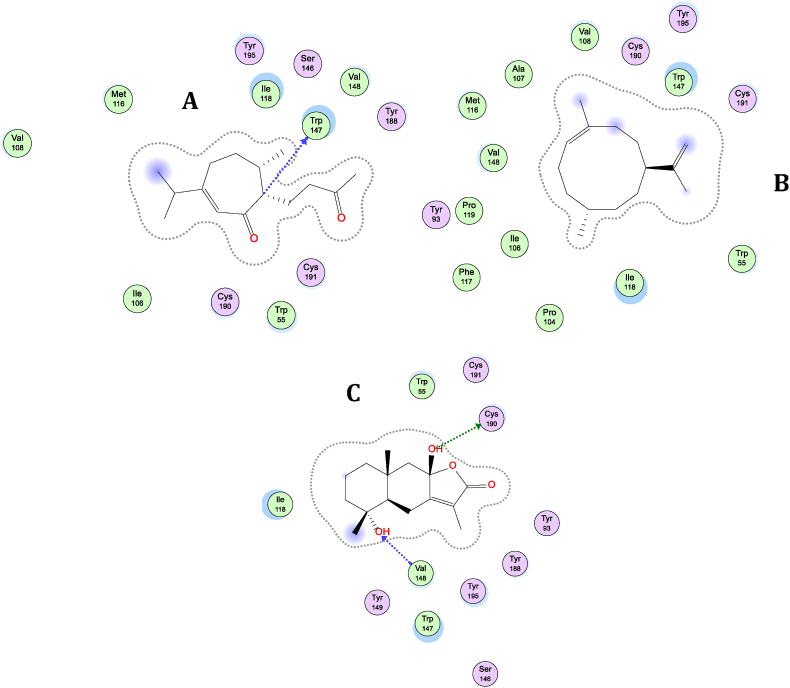
Molecular interactions between the chosen compounds and nAChRÿ3, illustrated in (A) Dihydroxyeudesmene with nAChRÿ3, (B) Gibberodione with nAChRÿ3, and (C) Germacrene-E with nAChRÿ3.

We employed BIOVIA Discovery Studio Visualizer v 2019 Client (BIOVIA, San Diego, CA, USA) to visualize the molecular interactions involving the selected compounds (BIOVIA, 2005). In this section, we depict the molecular interactions of nAChRÿ3 with the top three compounds, namely Dihydroxyeudesmene, Gibberodione, and Germacrene-E. The most favorable interaction is observed with the Dihydroxyeudesmene compound, which binds to the active site residue of nAChRÿ3. This interaction involves hydrogen bonds with VAL-148, TYR-195, CYS-190, and VAL-148. Furthermore, substantial hydrophobic interactions are formed between the ligand and various residues, including CYS-190, CYS-191, ILE-118, TYR-93, TRP-147, TYR-188, and TYR-195. These hydrophobic interactions are facilitated through stacked Alkyl and Pi-Alkyl bonds (Table 1 and Fig. 2A).

**Table 1:** Data for the molecular docking of sesquiterpene compounds obtained from Cinnamomum with the human nicotinic acetylcholine receptors (nAChRs) subtype nAChRÿ3 (4ZK4).

Compound	ΔG (kcal/mol)	Ki (μM)	Amino Acid Residue	
			Hydrogen Bond	Hydrophobic
Gibberodione	−8.56	0.53		**Pi-Sigma:** TRP147; **Alkyl:** ILE118, CYS190, CYS191, CYS190, VAL108, MET116, ILE118, CYS191, ILE118, VAL148; **Pi-Alkyl:** TRP55, TRP147, TYR188, TYR195
Germacrene-E	−8.56	0.58		**Pi-Sigma:** TRP147, TRP147; **Alkyl:** ILE118, VAL148, ILE118, VAL148, VAL108, VAL148, ILE118; **Pi-Alkyl:** TRP55, PHE117, TRP147, TRP147
Dihydroxyeudesmene	−8.50	0.59	VAL148, TYR195, CYS190, VAL148	**Alkyl:** CYS190, CYS191, ILE118, ILE118, CYS190, CYS191; **Pi-Alkyl:** TYR93, TRP147, TRP147, TYR188, TYR188, TYR195, TYR195
Copaene	−8.40	0.70		**Pi-Sigma:** TYR93, TYR188; **Alkyl:** ILE118, CYS190, CYS190, CYS191, ILE118, ILE118, CYS190, ILE118, VAL148; **Pi-Alkyl:** TRP55, TRP55, TRP55, TRP55, TRP55, TYR93, TRP147, TRP147, TRP147, TRP147, TRP147, TRP147, TYR195, TYR195
Bulnesene	−8.38	0.72		**Pi-Sigma:** TRP147; **Alkyl:** VAL108, ILE118, ILE118, VAL148, VAL148, CYS190, CYS191, ILE118, VAL148, VAL108, VAL148, ILE118; **Pi-Alkyl:** TRP55, TRP55, TRP147, TRP147, TRP147
Germacrene-B	−8.38	0.73		**Pi-Sigma:** TRP55; **Alkyl:** CYS190, CYS191, CYS190, CYS190, CYS191, ILE118, VAL148, VAL108, VAL148; **Pi-Alkyl:** TRP55, TRP147, TRP147, TRP147, TRP147, TYR188, TYR195, TYR195
Muurolene	−8.38	0.73		**Pi-Sigma:** TYR93, TRP147; **Alkyl:** ILE118, CYS190, CYS190, CYS191, CYS191, ILE118, CYS190; **Pi-Alkyl:** TRP55, TRP55, TRP55, TYR93, TRP147, TRP147, TRP147, TYR188, TYR188, TYR195, TYR195, TYR195
Bisabolene	−8.31	0.80		**Pi-Sigma:** TYR195, TRP147, TRP147; **Alkyl:** ILE118, VAL108, MET116, ILE118, ILE118, VAL148; **Pi-Alkyl:** TRP55, TRP55, TYR93, TRP147, TYR188, TYR188, TYR195
Cubebene	−8.31	0.81		**Pi-Sigma:** TRP147; **Alkyl:** VAL108, ILE118, VAL148, VAL148, VAL148, CYS190, CYS191, ILE118, MET116, ILE118, VAL108, VAL148, ILE118, VAL148, ILE118; **Pi-Alkyl:** TRP55, PHE117, TRP147, TRP147, TRP147, TRP147, TRP147
Cadienene	−8.30	0.82		**Alkyl:** VAL108, ILE118, ILE118, VAL148, CYS190, CYS191, CYS191, ILE118, CYS190, VAL108, MET116, ILE118, VAL148, ILE118, VAL148; **Pi-Alkyl:** TRP147, TRP147, TRP147, TYR195
Hydroxy-T-Murolol	−8.29	0.84	TYR195, ILE106, VAL148, ILE118	**Pi-Sigma:** TRP147; **Alkyl:** ILE118, VAL148, VAL148, CYS190, CYS191, ILE118, CYS190, CYS191, ILE118; **Pi-Alkyl:** TRP147, TRP147, TRP147, TYR195
Bisabolol	−8.28	0.86	TRP147	**Pi-Sigma:** TYR188; **Alkyl:** ILE118, VAL148, VAL108, VAL148; **Pi-Alkyl:** TRP55, TYR93, TYR93, TRP147, TRP147, TRP147
Cadinol	−8.28	0.85	ILE118	**Pi-Sigma:** TYR188; **Alkyl:** ILE118, VAL148, CYS190, CYS191, CYS191, VAL108, VAL148, CYS190, CYS191; **Pi-Alkyl:** TYR93, TRP147, TYR188, TYR195, TYR195
Germacrene-D	−8.26	0.88		**Pi-Sigma:** TYR195; **Alkyl:** ILE118, VAL148, CYS191, CYS191, ILE118, CYS190, ILE118, VAL148, ILE118, VAL148; **Pi-Alkyl:** TRP55, PHE117, TRP147, TRP147, TRP147
Germacrene-C	−8.18	1.02		**Alkyl:** CYS190, ILE118, CYS190, CYS190, CYS191, ILE118, CYS191, ILE118, VAL148; **Pi-Alkyl:** TRP55, TRP55, TRP147, TRP147, TYR188, TYR195, TYR195
Germacrene-A	−8.14	1.07		**Pi-Sigma:** TRP55; **Alkyl:** CYS190, CYS190, CYS191, CYS190, MET116, ILE118, ILE118, VAL148; **Pi-Alkyl:** TRP55, TRP147, TRP147, TRP147, TYR188, TYR195, TYR195
Eudesmene	−8.10	1.15	TRP147	**Alkyl:** CYS190, CYS191, ILE118; **Pi-Alkyl:** TRP55, TRP55, TRP55, TRP55, TRP147, TRP147, TRP147, TRP147, TRP147, TRP147, TYR188, TYR195, TYR195
Cedrene	−8.05	1.26		**Pi-Sigma:** TYR195, TYR93, TYR188; **Alkyl:** ILE118, CYS190, CYS191, CYS190, CYS191, ILE118, CYS190; **Pi-Alkyl:** TRP55, TRP55, TRP55, TRP55, TRP55, TYR93, TYR93, TRP147, TRP147, TRP147, TRP147, TYR188, TYR188, TYR195
Spathulenol	−8.03	1.30	MET116	**Alkyl:** ILE118, VAL148, CYS191, ILE118, VAL148, ILE118, VAL108, VAL148, VAL108, MET116, CYS191; **Pi-Alkyl:** TRP147, TRP147, TRP147, TRP147, TRP147, TYR195
Oxyphyllonediol-B	−8.02	1.32	TRP147, TYR195, TRP147	**Alkyl:** CYS190, CYS191, ILE118; **Pi-Alkyl:** TRP55, TRP147, TRP147, TYR188, TYR195
Curcumene	−7.91	1.58		**Pi-Pi T-shaped:** TYR93, TRP147, TRP147; **Alkyl:** TYR195, ILE118, CYS190, VAL108, MET116, ILE118, VAL108, ILE118, VAL148; **Pi-Alkyl:** TRP55, TRP55, TYR93, TRP147, TYR188, TYR195
Calamanene	−7.88	1.67		**Pi-Sigma**: ILE118, TRP147; **Pi-Pi T-shaped:** TRP147; **Alkyl:** ILE118, VAL148, CYS191, ILE118, ILE118, VAL148, MET116, ILE118; **Pi-Alkyl:** TRP55, TRP55, TRP147, TRP147, CYS190, CYS191
Calacorene	−7.85	1.76		**Pi-Sigma**: TYR195, TYR93; **Pi-Pi T-shaped:** TRP147; **Alkyl:** CYS190, CYS190, CYS191, ILE118, VAL148; **Pi-Alkyl:** TRP55, TRP55, TYR93, TRP147, TRP147, TRP147, TRP147, TRP147, TRP147, TYR188, TYR188, TYR195, TYR195, ILE118, CYS190, CYS191
Oxyphyllenodiol-A	−7.81	1.87	ILE118, VAL148, VAL148, TYR195, PHE117	**Alkyl:** VAL148, ILE118, ILE118, CYS190, CYS190; **Pi-Alkyl:** TRP147, TRP147, TRP147, TYR195
Isoledene	−7.78	1.97		**Pi-Sigma**: TRP147, TYR188; **Alkyl:** CYS190, CYS190, CYS191, ILE118, ILE118, CYS190, CYS190, CYS191; **Pi-Alkyl:** TRP55, TRP55, TRP55, TRP55, TRP55, TYR93, TYR93, TRP147, TRP147, TRP147, TRP147, TRP147, TYR188, TYR195, TYR195, TYR195
Caryolane	−7.76	2.04	ILE118	**Alkyl:** CYS190, CYS191, ILE118, VAL148, ILE118, CYS190, CYS191; **Pi-Alkyl:** TRP55, TYR93, TRP147, TRP147, TYR188, TYR188
Caryophyllene	−7.76	2.06		**Pi-Sigma**: TYR188; **Alkyl:** CYS190, ILE118, ILE118, VAL148, ILE118, CYS191, CYS190, CYS191, ILE118, CYS190; **Pi-Alkyl:** TRP55, TRP55, TRP147, TRP147, TRP147, TRP147, TRP147, TYR195, TYR195
Patchouli-Alcohol	−7.71	2.24	TRP147, TRP147	**Pi-Sigma**: TRP147, TYR93, TYR188; **Alkyl:** CYS190, CYS190, CYS191, ILE118, CYS190, CYS191, ILE118, CYS190; **Pi-Alkyl:** TRP55, TRP55, TRP55, TRP55, TRP55, TRP55, TYR93, TRP147, TRP147, TRP147, TYR188, TYR188, TYR188, TYR195, TYR195
Apocynol-A	−7.66	2.42	VAL148, TYR195, ILE118, VAL148	**Pi-Sigma**: TRP147, TRP147; **Alkyl:** CYS190, ILE118; **Pi-Alkyl:** TRP55, TRP55, TRP55, TRP55, TRP147, TRP147
Humulene	−7.64	2.49		**Pi-Sigma**: TYR195, TRP55; **Alkyl:** ILE118, CYS190, CYS191, MET116, ILE118, CYS191, VAL148, CYS190, CYS191, ILE118, CYS190; **Pi-Alkyl:** TRP55, TRP147, TRP147, TRP147, TYR188, TYR195
Blumenol-C	−7.58	2.77	ILE118, ILE118	**Pi-Sigma**: TRP147; **Alkyl:** ILE118, CYS190, CYS190, CYS191; **Pi-Alkyl:** TRP55, TRP55, TRP55, TRP55, TRP147, TRP147, TYR195
Megastigmatrienol	−7.46	3.43		**Pi-Sigma**: TRP55, TRP147, TRP147, TRP147; **Alkyl:** ILE118, CYS190, CYS191, VAL148, ILE118, VAL108, ILE118, VAL148; **Pi-Alkyl:** TRP55, TRP147, TRP147, TRP147, TYR195, TYR195
Tanacetene	−7.42	3.65		**Pi-Sigma**: TYR93; **Alkyl:** ILE118, VAL148, VAL108, VAL148, ILE118, CYS190, CYS191; **Pi-Alkyl:** TRP55, TYR93, TRP147, TRP147, TRP147, TRP147, TRP147
Apocynol-B	−7.38	3.87	TRP147, ILE118	**Pi-Sigma**: TRP147, TRP147, TRP147, TRP147; **Alkyl:** ILE118; **Pi-Alkyl:** TRP55, TRP55, TRP147
Aromadendrane	−7.35	4.12	ILE118	**Pi-Sigma**: TRP147, TYR195, TYR188; **Alkyl:** CYS190, CYS191, ILE118; **Pi-Alkyl:** TRP55, TYR93, TRP147, TRP147, TRP147, TYR188, TYR188, TYR195
Blumenol-A	−7.35	4.07	TRP147, TYR195, VAL148	**Pi-Sigma**: TRP147, TYR188, TYR195; **Alkyl:** ILE118; **Pi-Alkyl:** TRP55, TYR93, TRP147, TRP147, TYR188, TYR188
Boscialin	−7.34	4.18	TRP147, TYR93	**Pi-Sigma**: TRP55, TRP147; **Alkyl:** CYS190, CYS191, CYS190, CYS191; **Pi-Alkyl:** TRP55, TYR93, TRP147, TYR188, TYR195, TYR195
Dehydrovomofoliol	−7.33	4.25	TRP147	**Pi-Sigma**: TRP147, TYR188; **Alkyl:** ILE118, CYS190; **Pi-Alkyl:** TRP55, TRP55, TRP55, TYR93, TYR93, TRP147
Oxonerolidol	−7.27	4.67	TRP147	**Pi-Sigma**: TRP147; **Alkyl:** ILE118, VAL108, VAL148; **Pi-Alkyl:** TRP147
Wilsonol-C	−7.24	4.90	ILE118, TRP147, TRP147, TRP147	**Alkyl:** VAL148, ILE118, CYS190, CYS191, ILE118; **Pi-Alkyl:** TRP55, TRP55, TRP147, TRP147, TYR195
Alpinenone	−7.16	5.64	TYR195, PHE117	**Alkyl:** ILE118, VAL148, VAL108, VAL148, CYS190, CYS191, MET116, CYS191; **Pi-Alkyl:** TRP147, TYR195
Wilsonol-H	−7.14	5.82	TYR195, TRP147	**Pi-Sigma**: TRP55, TYR195; **Alkyl:** CYS190, ILE118; **Pi-Alkyl:** TRP55, TRP55, TRP147, TRP147, TYR188
Blumenol-B	−7.06	6.70	TRP147, VAL148, TYR195, VAL148	**Pi-Sigma**: TRP55, TYR188, TRP147; **Alkyl:** ILE118; **Pi-Alkyl:** TRP55, TYR93, TRP147, TRP147, TYR188, TYR195
Megastigmane	−7.03	7.08	TRP147, TYR195, VAL148	**Pi-Sigma**: TYR195, TYR195, TRP147, TRP147; **Alkyl:** CYS190, CYS191; **Pi-Alkyl:** TYR188
Wilsonol-B	−7.01	7.31	PRO104, TRP147, TYR195, TRP147, TRP147	**Alkyl:** VAL108, VAL148, VAL108, MET116, ILE118, ILE118; **Pi-Alkyl:** TRP147, TRP147
Wilsonol-E	−6.83	9.94	ILE118, TRP147, TRP147, ILE106, ILE106, TRP147	**Pi-Sigma**: TRP147; **Alkyl:** VAL108, VAL148, VAL108, MET116, CYS191, ILE118; **Pi-Alkyl:** TRP147
Wilsonol-A	−6.80	10.29	TYR195, TRP147, VAL148	**Pi-Sigma**: TYR195, TYR195; **Alkyl:** CYS190, ILE118, CYS190; **Pi-Alkyl:** TRP55, TRP55, TYR188
Lasianthiosonide-C	−6.69	12.44	ARG79, ILE118, ILE118, ILE106, ILE118, TRP147, PHE117	**Alkyl:** VAL108, CYS191, CYS191, VAL108, VAL148
Icariside-B1	−6.69	12.58	VAL148, TYR195, CYS191	**Pi-Sigma**: TYR195, TYR188; **Alkyl:** CYS190, CYS191, CYS190, CYS191; **Pi-Alkyl:** TYR188, TYR195, TYR195
Grasshopper-Ketone	−6.68	12.79	TRP147, TYR93, TRP147	**Pi-Sigma**: TRP55, TRP147, TYR195, TYR195; **Alkyl:** CYS190; **Pi-Alkyl:** TRP55, TRP55, TRP147, TYR188
Abscisic Acid	−6.58	14.92	TYR195, VAL148, TYR195, VAL148	**Pi-Sigma**: TRP55, TRP147, TYR188, TYR195; **Alkyl:** CYS190, CYS191, ILE118, VAL148; **Pi-Alkyl:** TRP55, TRP55, TYR93, TRP147, TRP147, TRP147, TYR188
Wilsonol-F	−6.55	15.76	TYR93, ILE118, SER146	**Pi-Sigma**: TRP147, TYR195, TYR93; **Alkyl:** CYS190, ILE118, CYS190, CYS191; **Pi-Alkyl:** TRP55, TRP55, TRP147, TYR188, TYR195
Lasianthiosonide-A	−5.39	112.26	ASP77, GLU153, VAL148, SER150, GLU153	**Alkyl:** MET116, CYS191, VAL108, MET116, VAL108
Lasianthiosonide-B	−5.33	123.56	ILE118, TRP147, SER150, ILE106, TRP147, TRP147, SER150, MET116	**Alkyl:** VAL108, MET116, VAL108, VAL108, CYS191

Gibberodione and Germacrene-E exhibit distinct interaction patterns when interacting with nAChRÿ3. Notably, no hydrogen bonds are formed during their interaction with nAChRÿ3. However, Gibberodione demonstrates the capacity to establish Pi-Sigma, Alkyl, and Pi-Alkyl interactions with several residues, including TRP-147, ILE-118, CYS-190, CYS-191, CYS-190, VAL-108, MET-116, ILE-118, CYS-191, ILE-118, VAL-148, TRP-55, TRP-147, TYR-188, and TYR-195. On the other hand, Germacrene-E forms a complex with TRP-147, ILE-118, VAL-148, VAL-108, and PHE-117, relying on hydrophobic interactions facilitated by Pi-Sigma, Alkyl, and Pi-Alkyl bonds (Fig. 2B and C).

### 3.4 Molecular dynamics (MD) simulations

**Fig. 3:**
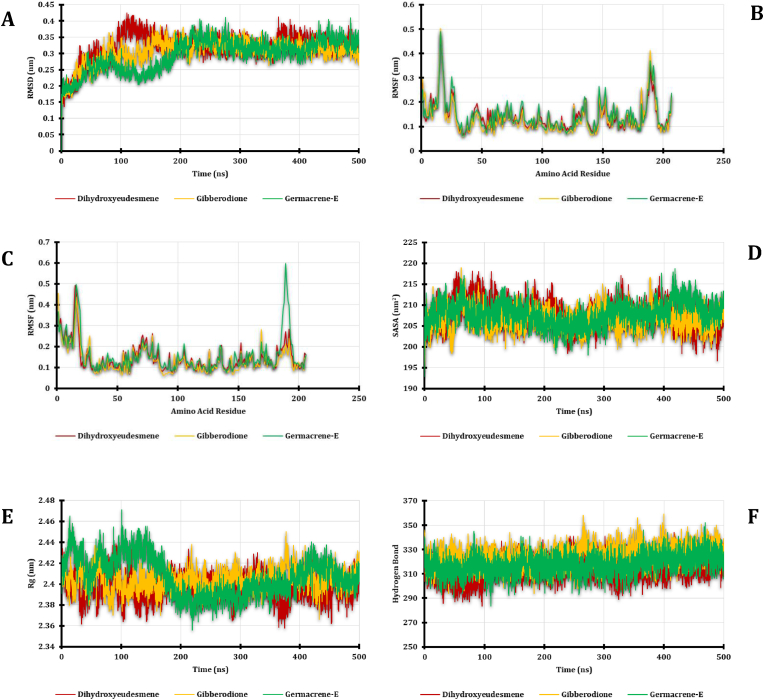
Molecular dynamics simulations were performed on nAChRÿ3, and various analyses were conducted, including: (A) Evaluation of RMSD (Root Mean Square Deviation); (B) Assessment of RMSF (Root Mean Square Fluctuations) for Chain-A; (C) Examination of RMSF (Root Mean Square Fluctuations) for Chain-B; (D) Analysis of SASA (Solvent Accessible Surface Area); (E) Determination of Rg (Radius of Gyration); and (F) Calculation of Hydrogen Bonding Energy.

To gain insights into the flexibility of the Dihydroxyeudesmene, Gibberodione, and Germacrene-E complexes during their interaction with nAChRÿ3, molecular dynamics simulations were conducted. The root mean square deviation (RMSD) of the atoms within these complexes was computed to assess their rigidity (Hikmawati et al., 2022). Initially, the Dihydroxyeudesmene complex displayed a higher RMSD trend, primarily due to its elevated level of flexibility. This analogous trend was also observed in the Gibberodione complex. However, all three complexes eventually reached a stable state after 200 ns, with Germacrene-E demonstrating optimal stability right from the start of the simulation and maintaining it throughout the entire 500 ns duration. Throughout the simulation period, all three systems exhibited some degree of deviation, yet they consistently retained a stable state. Additionally, it's worth noting that the RMSD profiles for all three systems remained below 4.0 Å, indicating the stability of these complexes (Fig. 3A).

Analysis of the solvent-accessible surface area (SASA) of the complex was conducted to gain insights into alterations in the protein's surface area. Higher SASA values indicate an expansion of the surface area, while lower SASA values suggest a reduction in the protein's volume (Muchtaridi et al., 2023). Throughout the simulation period, it was observed that the Dihydroxyeudesmene complex with nAChRÿ3 consistently exhibited a slightly higher SASA at the initial part of the simulation, when compared to the docked complexes with Gibberodione and Germacrene-E. This finding suggests that nAChRÿ3 forms a stable and rigid binding interaction with the Dihydroxyeudesmene ligand. The complex achieved a stable state after 300 ns and maintained its rigidity for the remainder of the simulation duration (Fig. 3D). Similarly, the nAChRÿ3 complex displayed a comparable trend, reducing the SASA profile upon binding with the ligand molecule. This reduction in SASA implies a more compact binding interaction between nAChRÿ3 and the ligand, indicative of a stable and well-defined complex.

The radius of gyration (Rg) profile was also calculated for the simulated system to assess the stability of the complex. The Rg pattern observed in the nAChRÿ3 system remained relatively constant, indicating a stable and less mobile nature of the complex (Fig. 3E). Consequently, the Dihydroxyeudesmene and Gibberodione complexes of nAChRÿ3 exhibited similar Rg values, signifying a comparable degree of compactness in the receptor-ligand complexes. On the other hand, the Germacrene-E complex with nAChRÿ3 displayed a lower Rg, suggesting a higher level of compactness in this particular receptor-ligand interaction. Additionally, the stability of the complex was influenced by the hydrogen bonding pattern within the protein system. The figure illustrates that the nAChRÿ3 complex maintained a stable trend in hydrogen bonding throughout the entire simulation period (Fig. 3F).

Additionally, we conducted an analysis of the entire complex using the root mean square deviation to gain insights into the flexibility of individual amino acid residues within the complex. In general, the RMSF values for the nAChRÿ3 complex remained below 3.0 Å, with the noteworthy exception of specific residues such as TRP-55, TYR-93, VAL-108, MET-116, ILE-118, SER-146, TRP-147, VAL-148, TYR-188, CYS-190, CYS-191, and TYR-195. This assessment also highlighted that the nAChRÿ3 complex exhibited lower RMSF profiles for the majority of its residues, underscoring the substantial stability of this complex, as visually represented in Fig. 3B and C. These findings underscore the overall inflexibility and consistent stability of the complex, further emphasizing the robust and dependable nature of the receptor-ligand interaction.

## 4 Discussions

A range of sesquiterpene derivative compounds derived from the Cinnamomum genus have been scientifically established as viable alternatives with the potential to inhibit human nicotinic acetylcholine receptors (nAChRs), particularly the nAChRÿ3 subtype, which serves as a pivotal receptor for nicotine. Additionally, sesquiterpenes have demonstrated the potential for inhibiting Acetylcholinesterase (AChE) based on various research studies. This research project is centered on elucidating the inhibitory effects on nAChRÿ3 by means of predicting the binding affinity of sesquiterpene phytochemicals sourced from the Cinnamomum genus. This is achieved through the application of in silico molecular docking investigations and molecular dynamics simulations. The nAChRÿ3 receptor, responsible for orchestrating the transmission of nicotine signals within the human nervous system, becomes the focal point of this inquiry. Consequently, the inhibition of nAChRÿ3 is anticipated to result in a diminished response to nicotine, thereby reducing the inclination to smoke. This could be instrumental in aiding individuals in their efforts to quit smoking (Ho et al., 2020; Mineur et al., 2023). Nicotine replacement therapy, employing sesquiterpene derivative compounds extracted from the Cinnamomum genus, emerges as a viable pharmacotherapeutic approach for smoking cessation. The fundamental premise of this therapy revolves around diminishing the urge to resume smoking after quitting. The recommended duration for nicotine replacement therapy typically spans around three months. This therapeutic approach is relatively safer when compared to electronic cigarettes, as it does not contain carcinogenic substances and other hazardous chemicals, prioritizing the well-being of individuals seeking to quit smoking.

In light of this issue, it's essential to recognize that prior to this research, no investigations had been conducted on nicotine replacement utilizing sesquiterpenes derived from the Cinnamomum genus or interaction tests with the nAChRÿ3 receptor. Therefore, this study stands at the forefront of scientific exploration, employing endemic Cinnamomum genus plants that can be subjected to extraction using a variety of methods. Consequently, several advantages inherent in these natural herbal plants of the Cinnamomum genus have been harnessed for their potential use in nicotine replacement therapy (NRT) (Taylor et al., 2021; Wang et al., 2022). Notably, ethnopharmacological studies have compiled a list of Cinnamomum genus plants exhibiting diverse pharmacological activities, underscoring their significance in the realm of conventional traditional medicine. However, it is worth noting that this research represents an innovative leap in the quest to uncover the potential of these natural resources as nicotine substitutes. To date, the full spectrum of possibilities presented by Cinnamomum genus plants as nicotine replacements remains largely unexplored, rendering this research a valuable addition to our scientific understanding of the health-related advantages offered by these plants. The opportunity to explore the benefits of these plants within the context of smoking cessation therapy could also pave the way for the development of safer and more effective medications designed to assist individuals in breaking free from their smoking habits.

The nAChRÿ3 structure has been effectively discerned through the utilization of X-ray diffraction, yielding a remarkable resolution of 1.90 Å. This receptor, comprising 229 amino acids, is subdivided into five distinct domains, denoted as Domain A, Domain B, Domain C, Domain D, and Domain E. Within this framework, the active residues of nAChRÿ3 are distributed across various domains, with a notable concentration within Domain C and Domain E, which establish direct interactions with 7-(5-isopropoxy-pyridin-3-yl)-1-methyl-1,7-diaza-spiro[4.4]nonane, the native ligand of nAChRÿ3. The native ligand, as detailed in the crystal structure of a chimeric acetylcholine-binding protein (Ac-AChBP) containing loop C from the human α3 nicotinic acetylcholine receptor, is a synthetic partial agonist. While it does not exhibit the full agonistic activity of nicotine, it binds effectively to the receptor, providing a critical model for studying receptor-ligand interactions. This synthetic compound mimics some aspects of the binding behavior of natural ligands, making it a valuable tool for structural and functional analyses.

The presence of high-resolution structural data underscores the substantial potential of nAChRÿ3 as a target for therapeutic development. The insights gained from the interaction of human nAChRÿ3 with this synthetic ligand have unveiled key aspects of inhibitor binding mechanics, which are essential for rational drug design. Moreover, nicotine, a natural full agonist of nAChRs, serves as a benchmark for evaluating the therapeutic efficacy of potential inhibitors targeting nAChRÿ3. These findings highlight the receptor's pivotal role in mediating the effects of smoking and its potential as a therapeutic target. The structural scrutiny of nAChRÿ3, along with its complexes involving both synthetic and natural ligands, provides a foundation for developing therapeutic candidates. Such therapies could serve as effective strategies for mitigating or halting thedeleterious effects associated with smoking, offering a significant advancement in nicotine addiction treatment.

In light of our discoveries, it is likely that our leading candidates establish binding interactions with the catalytic residues of the nAChRÿ3 targets. Consequently, Dihydroxyeudesmene experiences stabilization through the formation of multiple hydrogen bonds and hydrophobic interactions. As demonstrated in Table 1, hydrogen bonds were identified with the catalytic residues VAL-148, TYR-195, CYS-190, and VAL-148 in the nAChRÿ3-Dihydroxyeudesmene complex. In contrast, Gibberodione and Germacrene-E predominantly foster hydrophobic interactions at the binding active site of nAChRÿ3. Likewise, within the nAChRÿ3-Dihydroxyeudesmene complex, several amino acid residues, including CYS-190, CYS-191, ILE-118, TYR-93, TRP-147, TYR-188, and TYR-195 (Fig. 2A), play a pivotal role in catalysis, and the docking orientation closely mirrors that of the X-ray structural control involving (5R)-1-methyl-7-[5-(propan-2-yloxy)pyridin-3-yl]-1,7-diazaspiro[4.4]nonane. Moreover, the nAChRÿ3 receptor encompasses catalytic residues, such as TRP-55, TYR-93, VAL-108, MET-116, ILE-118, SER-146, TRP-147, VAL-148, TYR-188, CYS-190, CYS-191, and TYR-195. Similarly, Gibberodione establishes numerous hydrophobic interactions with the active site residues, namely TRP-147, ILE-118, CYS-190, CYS-191, CYS-190, VAL-108, MET-116, ILE-118, CYS-191, ILE- 118, VAL-148, TRP-55, TRP-147, TYR-188, and TYR-195. Furthermore, Germacrene-E also forms multiple hydrophobic interactions with residues at TRP-147, ILE-118, VAL-148, VAL-108, and PHE-117.

Molecular dynamics (MD) simulation represents a powerful technique for comprehending the stability and dynamics of protein-ligand interactions. When applied in the development of potential therapeutic agents, MD simulations aid in identifying structural deficiencies essential for creating novel compounds with enhanced target binding capabilities (Fakih, 2021; Hidayat and Fakih, 2021). The integration of MD simulations into the therapeutic candidate design process offers valuable structural insights and highlights the impact of protein structural stability on ligand binding. This approach not only enhances the accuracy of binding pose sampling but also refines affinity estimations, ultimately leading to greater structural precision (Huang et al., 2022). Increased values in metrics such as RMSD, RMSF, Rg, and SASA signify heightened flexibility within the system. Our MD simulations were conducted on the docking complex to explore how protein structure evolves when ligands are introduced. After 200 ns of simulation, the nAChRÿ3 complex reached a state of stability. The RMSD profiles for both systems consistently registered values below 4.0 Å, underscoring the stability of the complexes. Moreover, the SASA, Rg, H-Bond, and RMSF values (Fig. 3) further validate the complex's stability.

These computational analyses and statistical data contribute critical insights for the development of potential candidates in the context of Nicotine Replacement Therapy (NRT). Nonetheless, the efficacy and eventual release of new pharmaceuticals are contingent on considerations encompassing pharmacokinetics, efficacy, and safety profiles. Molecular docking analysis, particularly ADMET phytochemical analysis focused on compounds from the Cinnamomum genus, provides comprehensive insights into physicochemical properties, pharmacokinetics, and drug-related characteristics. Therefore, in-silico screening serves as an alternative approach for the identification of potential therapeutic agents from natural sources. In conclusion, our MD simulations identified Dihydroxyeudesmene, Gibberodione, and Germacrene-E as the most stable compounds, exhibiting the highest binding free energy, thereby emerging as promising candidates for further exploration in the realm of NRT development.

## 5 Conclusion

This study delves into the realm of sesquiterpene compounds, specifically investigating a subset of 55 compounds originating from the Cinnamomum genus, with the aim of inhibiting the activity of human nicotinic acetylcholine receptors (nAChRs), specifically the nAChRÿ3 subtype. The inhibition occurs through binding to the receptor's active sites, including TRP-55, TYR-93, VAL-108, MET- 116, ILE-118, SER-146, TRP-147, VAL-148, TYR-188, CYS-190, CYS-191, and TYR-195. The research employs molecular docking techniques to elucidate the intricate binding modes of the selected virtual candidates, offering a comprehensive understanding of their interactions with the receptor. These interactions encompass hydrogen bonding, van der Waals forces, and hydrophobic interactions. Impressively, the study reveals that the nAChRÿ3 receptor exhibits superior MM-GBSA values compared to the native ligand molecule, (5R)-1-methyl-7-[5-(propan-2-yloxy)pyridin-3-yl]-1,7-diazaspiro[4.4]nonane. Further insights are gleaned through molecular dynamics and simulation studies, indicating that Dihydroxyeudesmene, Gibberodione, and Germacrene-E form stable complexes with the nAChRÿ3 receptor, albeit via distinct conformational changes. Consequently, these compounds emerge as promising therapeutic candidates for Nicotine Replacement Therapy (NRT). However, it is essential to underscore that further experimentation and validation within wet laboratories are imperative to progress towards the development of effective and improved therapies employing these phytochemicals for smoking cessation.

## CRediT authorship contribution statement

**Taufik Muhammad Fakih:** Conceptualization, Methodology, Formal analysis, Writing – original draft, Visualization. **Aden Dhana Rizkita:** Supervision, Project administration, Resources, Writing – review & editing. **Sintia Ayu Dewi:** Data curation, Software, Investigation, Validation, Writing – review & editing. **Muchtaridi Muchtaridi:** Funding acquisition, Supervision, Writing – review & editing, All authors have read and approved the final manuscript and agree to be accountable for all aspects of the work.

## Funding

The 10.13039/501100023174Ministry of Education, Culture, Research, and Technology (Kemdikbudristek) of the Republic of Indonesia, through the 2023 Penelitian Dosen Pemula grant program outlined under grant No. 180/E5/PG.02.00.PL/2023.

## Declaration of competing interest

The authors declare that they have no known competing financial interests or personal relationships that could have appeared to influence the work reported in this paper.

## Data Availability

No data was used for the research described in the article.
